# Development and Standardization of a Furosemide Stress Test to Predict the Severity of Acute Kidney Injury

**DOI:** 10.1186/cc13015

**Published:** 2013-09-20

**Authors:** Lakhmir S Chawla, Danielle L Davison, Ermira Brasha-Mitchell, Jay L Koyner, John M Arthur, Andrew D Shaw, James A Tumlin, Sharon A Trevino, Paul L Kimmel, Michael G Seneff

**Affiliations:** 1 Department of Anesthesiology and Critical Care Medicine, George Washington University Medical Center, 900 23rd street, Washington DC, 20037, USA; 2 Division of Renal Diseases and Hypertension, Department of Medicine, George Washington University Medical Center, 2150 Pennsylvania Avenue, Washington DC, 20037, USA; 3 Section of Nephrology, Department of Medicine, University of Chicago, 5841 South Maryland Avenue, Chicago, IL, 60637, USA; 4 829 CSB Division of Nephrology, Department of Medicine, Medical University of South Carolina, 96 Jonathan Lucas, Charleston, SC, 250623, USA; 5 Department of Anesthesiology, Duke University/Durham VAMC, Durham, DUMC 3094, Durham, NC, 27710, USA; 6 Renal Division, University of Tennessee College of Medicine at Chattanooga, 251 North Lyerly Street, Chattanooga, TN, 37404, USA; 7 Division of Kidney, Urologic, and Hematologic Diseases, National Institute of Diabetes, Digestive, and Kidney Diseases, NIH, 6707 Democracy Blvd, Bethesda, MD, 20817, USA

## Abstract

**Introduction:**

In the setting of early acute kidney injury (AKI), no test has been shown to definitively predict the progression to more severe stages.

**Methods:**

We investigated the ability of a furosemide stress test (FST) (one-time dose of 1.0 or 1.5 mg/kg depending on prior furosemide-exposure) to predict the development of AKIN Stage-III in 2 cohorts of critically ill subjects with early AKI. Cohort 1 was a retrospective cohort who received a FST in the setting of AKI in critically ill patients as part of Southern AKI Network. Cohort 2 was a prospective multicenter group of critically ill patients who received their FST in the setting of early AKI.

**Results:**

We studied 77 subjects; 23 from cohort 1 and 54 from cohort 2; 25 (32.4%) met the primary endpoint of progression to AKIN-III. Subjects with progressive AKI had significantly lower urine output following FST in each of the first 6 hours (p<0.001). The area under the receiver operator characteristic curves for the total urine output over the first 2 hours following FST to predict progression to AKIN-III was 0.87 (p = 0.001). The ideal-cutoff for predicting AKI progression during the first 2 hours following FST was a urine volume of less than 200mls(100ml/hr) with a sensitivity of 87.1% and specificity 84.1%.

**Conclusions:**

The FST in subjects with early AKI serves as a novel assessment of tubular function with robust predictive capacity to identify those patients with severe and progressive AKI. Future studies to validate these findings are warranted.

## Introduction

Acute kidney injury (AKI) is a clinical syndrome that is associated with significant morbidity and mortality [1,2]. The incidence of AKI has more than doubled in the past decade and is projected to continue to increase [3]. Patients with AKI are cared for by a multitude of specialists including, but not limited to: emergency medicine physicians, internists, pediatricians, surgeons, intensivists, and nephrologists [4]. Patients who develop AKI often require renal replacement therapy (RRT), but clinicians often disagree about the optimal timing of the initiation of RRT. During the Acute Kidney Injury Network (AKIN) multi-disciplinary consensus meeting, the question that was ranked highest was: 'When should RRT be initiated?' [4]. RRT is an invasive procedure with inherent risks, and one would not want to initiate this therapy if the patient were destined to recover renal function without intervention. However, a more conservative approach of initiating RRT late in the course of the AKI can subject the patient to adverse consequences [5]. Thus, if a test could be devised that predicts the likelihood of progressing to a more severe stage of AKI, decisions regarding optimal timing of RRT initiation would be better informed.

Because serum creatinine and oliguria are often late signs of significant AKI, more sensitive diagnostic tests are required [6-9]. This clinical need has led to the development of multiple candidate AKI biomarkers [6,8-10]. Because AKI biomarker levels change over time depending on the timing and severity of injury [9], a functional assessment of renal function might enhance biomarker performance. Since most common form(s) of intrinsic AKI involve acute tubular injury, we sought to develop a functional assessment of renal tubular function. Furosemide, a loop diuretic, has pharmacokinetic properties that make it an appealing functional tool. In contrast to other drugs cleared by the kidney, furosemide is not effectively filtered by the glomerulus. As an organic acid, furosemide is tightly bound to serum proteins and gains access to the tubular lumen by active secretion via the human organic anion transporter (hOAT) system in the proximal convoluted tubule [11,12]. Once in the tubular lumen, furosemide inhibits luminal active chloride transport throughout the thick ascending limb of Henle, thereby preventing sodium reabsorption and resulting in natriuresis and increased urine flow [13-15]. We surmised that furosemide-induced increases in urine output might be a method to assess the integrity of renal tubular function in the setting of early AKI. Specifically, we hypothesized that the kidney's response or lack of response to a furosemide challenge, as a clinical assessment of tubular function, could identify patients with severe tubular injury before it was clinically apparent (for example, a rise in creatinine). We sought to develop and standardize a furosemide stress test (FST) for patients with AKI and describe its performance characteristics.

## Materials and methods

We assembled two separate cohorts of critically ill patients with either stage I or II AKIN criteria (Additional file 1 - Table S1) [16], who were given a standardized dose of furosemide, and assessed their response and outcomes.

### Cohort 1

The Southern Acute Kidney Injury Network (SAKInet) [17] was formed in 2007 to collect samples from patients who developed AKI, with the goal of testing the diagnostic and prognostic accuracy of previously described and novel AKI biomarkers. For each subject, informed consent was obtained in accordance with The George Washington University Institutional Review Board-approved SAKInet protocol. We identified a subset of patients from the SAKInet cohort at the George Washington University who fulfilled the study criteria.

### Cohort 2

The protocol for cohort 2 was registered in clinicaltrials.gov. The study was carried out at the George Washington University (NCT00673244) and at the University of Chicago (NCT01275729). The respective university IRBs approved the identical protocol. Patients or their surrogates were required to sign informed consent prior to study entry. Patients were enrolled from June 2009 through December 2012. Urine sediment was assessed with the George Washington Urine Sediment Score (GW USS) as described previously [18]. Briefly, patients with a GW USS ≥2 have evidence of granular or epithelial cell casts in their urine sediment.

### Study criteria (both cohorts 1 and 2)

Inclusion criteria: (1) age greater than 18 years, admitted in an ICU; (2) AKIN stage I (6 hours of oliguria (<0.5 ml/kg/hour) or 0.3 mg/dL increase in serum creatinine or increase of 150 to 200% above baseline serum creatinine), or AKIN stage II (12 hours of oliguria (<0.5 ml/kg/hour) or increase of 200 to 300% above baseline serum creatinine); (3) indwelling bladder catheter; (4) presence of granular or epithelial cell casts on urine sediment (defined by GW USS ≥2), or fractional excretion of sodium (FeNa) >1.0%; and (5) patient deemed by the treating clinical team to be well-resuscitated.

Exclusion Criteria: (1) baseline estimated glomerular filtration rate (eGFR) <30 ml/minute/1.73m^2^; (2) history of renal allograft; (3) known pregnancy; (4) evidence of obstructive uropathy (for example, hydro-ureter); (5) evidence of active bleeding; (6) patients with allergy or known sensitivity to loop diuretics; (7) achievement of AKIN stage III criteria; or (8) evidence of volume depletion at the time of furosemide administration.

### Study procedures (cohort 1)

Patients in the SAKInet cohort who met the study criteria, and who received a furosemide dose of 1.0 mg/kg were entered into cohort 1. Replacement fluid was not protocolized in this group of subjects. Demographic and clinical data, urine sediment scores, and outcome data were abstracted from the case report forms.

### Study procedures (cohort 2)

Prior to FST urine was collected and scored with the GW USS [18]. Urinalysis was performed at each site (EBM-GW, JLK-UC). A pre-FST FeNa was only available if the treating team had ordered it for clinical purposes. After acquisition of informed consent, patients who were loop-diuretic naïve were given 1.0 mg/kg of intravenous furosemide. Because patients who were previously treated with loop diuretics within the previous 7 days were likely to have a blunted response over time compared to naïve patients, this group received an intravenous dose of 1.5 mg/kg (as little as 6 to 8 days of chronic loop-diuretic therapy is associated with a blunted response to furosemide due to increased distal tubular uptake of sodium in the thiazide-sensitive nephron segment)[19]. In order to minimize the risk of hypovolemia, urine output was replaced ml for ml each hour with either Ringers lactate or normal saline for six hours after the FST. The treating team could elect not to replace the volume if net volume loss was considered clinically desirable. Urine output was measured hourly for six hours and in total for 24 hours. Any and all adverse events related to furosemide were recorded including, but not limited to, tinnitus, hypokalemia, hypomagnesemia and hypotension. Patients were followed for 14 days or hospital discharge, whichever occurred first.

### Outcomes

The primary outcome was the progression to AKIN stage III (need for RRT, increase in serum creatinine of 300% over baseline, urine output of 0.3 cc/kg/hour × 24 hours) within 14 days of FST. The secondary outcome was the composite of achieving stage AKIN III or death within 14 days of the FST.

### Statistics

We assessed the distribution of demographic and clinical variables. Differences between proportions of patients with certain characteristics were assessed with the chi-square, Fisher exact, Student *t*, and Mann-Whitney tests as appropriate. The primary analysis was to assess the urine output response to the FST, which was determined by assessing the area under the curve (AUC) receiver operating characteristics (ROC) comparing the primary endpoint of progression to AKIN stage III and the secondary endpoint of death/AKIN III within 14 days of the FST. Multivariable logistic regression was used to create three models. Model 1 is a clinical model using the Acute Physiology and Chronic Health Evaluation (APACHE II) score, baseline urinary flow rate (UFR), baseline eGFR, and AKIN stage II at study entry. Model 2 has all univariate variables with a difference <0.10 entered as covariates. Model 3 is multivariate, backward elimination, logistic regression. All means are reported + standard error (SE) unless otherwise specified. Statistical analysis was performed using SPSS 18.0 (Chicago, Ill). Methodology used to calculate FeNa, APACHE II score[20], and cardiovascular (CV) SOFA[21] score and eGFR[22] is shown in Additional file 1.

## Results and discussion

**Figure 1:**
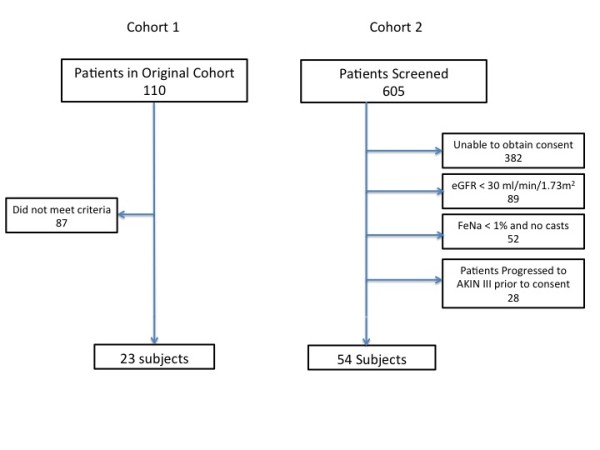
**Patient flow**. eGFR, estimated glomerular filtration rate; FeNa, fractional excretion of sodium; AKIN, Acute Kidney Injury Network.

We assessed a total of 77 patients, 23 patients from cohort 1 and 54 from cohort 2 (Figure 1). The mean age was 65.3 ± 1.6 years; 42.8% were male: Among the patients 44 (57.1%) were African-American, 23 (29.9%) were Caucasian, and 10 (13%) were Hispanic (Table 1). Of the 77 patients, 25 (32.4%) met the primary outcome of AKIN Stage III and 16 (20.7%) died. Of the total cohort, 32 (41.5%) met the secondary composite endpoint of AKIN III or death within 14 days of the FST. Of the 25 patients who progressed to AKIN stage III, 11 (44.0%) received RRT.

**Table 1:** Patient characteristics

Variable	Combined	Non-progressors	AKIN III	*P*
	*n *= 77	*n *= 52	*n *= 25	
**Age, years**	65.3 (1.6)	63.8 (2.2)	68.2 (1.9)	0.13
**Gender, % male**	42.8%	36.5%	56.0%	0.14
**Race, n (%)**				
African American	44 (57.1%)	29 (55.6%)	15 (60.0%)	0.63
Caucasian	23 (29.9%)	15 (28.8%)	8 (32.0%)	0.92
Hispanic	10 (13.0%)	8 (15.4%)	2 (8.0%)	0.48
**Comorbidities, n (%)**				
CKD	24 (31.0%)	17 (32.7%)	7 (28.0%)	0.80
Hypertension	60 (78.0%)	41 (78.8%)	19 (76.0%)	0.78
CHF	25 (33.0%)	15 (29.0%)	10 (40.0%)	0.44
DM	35 (44.0%)	22 (41.5%)	13 (52.0%)	0.47
**Nephrotoxic exposure, n (%)**				
NSAIDS	8 (10.0)%	6 (2.0%)	2 (1.0%)	1.00
Aminoglycosides	1 (1.0%)	0 (0.0%)	1 (0.4%)	0.63
Amphotericin	2 (3.0%)	2 (4.0%)	0 (0.0%)	1.00
Contrast	21 (27.0%)	15 (28.8%)	6 (23.1%)	0.79
Post-cardiac surgery	9 (11.7%)	6 (11.5%)	3 (12.0%)	1.00
Sepsis	15 (19.5%)	12 (23.1%)	3 (12.0%)	0.36
**Clinical Data**				
Baseline eGFR, ml/minute/1.73m^2^	68.6 (4.1)	60.0 (8.8)	73.3 (4.2)	0.15
Baseline UFR (ml/hr)	74.6 (11.6)	95.7 (16.3)	29.7 (4.2)	0.001
Furosemide-naïve, n (%)	29 (37.7%)	23 (44.2%)	6 (24.0%)	0.13
Urine cast score)	2.3 (0.13)	2.1 (0.16)	2.7 (0.23)	0.05
FeNa above 1%, n (%)^a^	14 (18.0%)	10 (19.2%)	4 (16.0%)	1.00
CV SOFA score	1.16 (0.3)	1.05 (0.2)	1.5 (0.4)	0.37
APACHE II score	17.8 (1.11)	16.5 (1.2)	21.6 (2.5)	0.08
**AKIN stage at enrollment, n (%)**				
AKIN I	41 (53.2%)	34 (65.4%)	7 (28.0%)	0.003
AKIN II	36 (46.7%)	18 (34.6%)	18 (72.0%)	0.003
**Outcomes, n (%)**				
Death	16 (20.7%)	7 (13.4%)	9 (36.0%)	0.04
**AKIN stage III**	25 (32.4%)	N/A	25 (100%)	N/A
**RRT**	11 (14.2%)	N/A	11 (44.0%)	N/A
**Death/AKIN III**	32 (41.5%)	7 (13.4%)	25 (100.0%)	0.001

Data are presented as mean ± standard error unless otherwise indicated. CKD, chronic kidney disease; CHF, congestive heart failure; DM, diabetes mellitus; NSAIDs, non-steroidal anti-inflammatory drugs; eGFR, estimated glomerular filtration rate; UFR, urinary flow rate; CV SOFA, Cardiovascular Sequential Organ Failure Assessment; APACHE, Acute Physiology and Chronic Health Evaluation; AKIN, Acute Kidney Injury Network; FeNa, fractional excretion of sodium; RRT, renal replacement therapy; RPP, renal perfusion pressure; n, number of patients; N/A, not applicable. ^a^FeNa not assessed in 29 patients because the George Washington University Urine Sediment Score was already ≥2 at the time of assessment.

In the overall cohort, 24 patients (31%) had chronic kidney disease (CKD). The numbers of patients with diabetes mellitus (DM), hypertension (HTN), and congestive heart failure (CHF) were 35 (44%), 60 (78%), and 25 (33%), respectively. The proportion of patients with CKD, HTN, CHF, and DM was not statistically different between progressors and non-progressors (Table 1). There was no difference in the prevalence of sepsis or recent cardiac surgery in those who did and did not progress. Baseline serum albumin concentration was not different between progressors and non-progressors (2.82 g/dL versus 2.89 g/dL, *P *= 0.89). Baseline serum lactate concentrations were not different between those who did and did not progress (data not shown). The mean cardiovascular Sequential Organ Failure Assessment (SOFA) score was 1.16 (0.3) and the mean APACHE II score was 17.8 (1.11); there was no difference between progressors and non-progressors (Table 1).

The baseline UFR for the 6 hours before the FST was 74.2 (11.6) ml/hour. The baseline UFR was 95.7 (16.3) and 29.7 (4.2) in the non-progressor group and in the progressor group, respectively (*P *<0.01). We assessed the capacity of the UFR in absolute values (UFR-raw), the UFR corrected for ideal body weight (UFR-IBW), and the UFR corrected for actual body weight (UFR-ABW) to predict progression to AKIN stage III. The ROC AUC for UFR-raw, UFR-IBW, and UFR-ABE was 0.76 (0.09), 0.71 (0.08), and 0.76 (0.08), respectively (Additional file 1 - Table S2). Within the combined cohort of patients, 36 (46.8%) had AKIN stage II by either urine output (UO) or serum creatinine (Scr) criteria at time of enrollment. There were fewer patients with AKIN stage II amongst non-progressors (*n *= 18 (34.6%)) compared to progressors (*n *= 18 (72%)) (*P*<0.003). In the combined cohort, the mean cast score was 2.3 (0.13). Non-progressors had a GW USS of 2.1 (0.16) compared to progressors who had a mean GW USS of 2.7 (0.23) (*P *= 0.05). The ROC AUC for GW USS to predict AKIN III was 0.63 (0.07). Patient characteristics of progressors and non-progressors are shown in Table 1. Patient characteristics in cohorts 1 and 2 are shown in Additional file 1 - Table S3.

FST urine output (for each increase of 10 ml of UO) was predictive of non-progression to AKIN stage III when baseline patient imbalances were placed into a multivariate logistic regression analysis (odds ratio (OR) 0.98, 95% CI 0.96, 0.99, *P *= 0.05). Multivariable logistic analyses are shown in Additional file 1 - Table S4.

### Furosemide stress test characteristics

**Figure 2:**
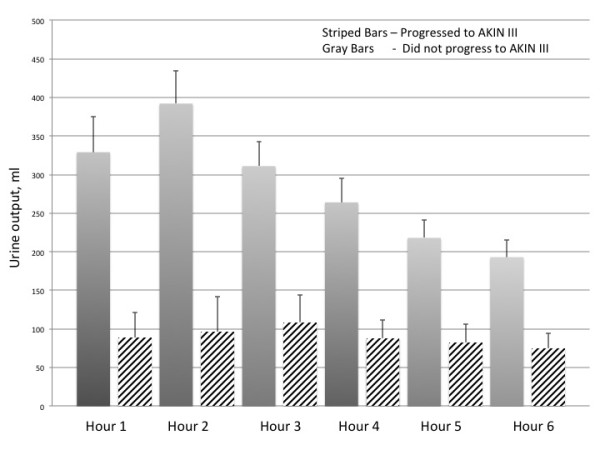
**Urinary output in response to furosemide stress test**.

The FST was well-tolerated with no episodes of hypotension or any other adverse event deemed attributable to the test. We assessed the UFR in response to furosemide. The maximum UFR was within the first 2 to 3 hours (Table 2, Figure 2). We compared the UFR in response to FST between those patients that progressed and did not progress to AKIN stage III (Table 2, Figure 2). For each hourly interval, progressors had a lower UFR response compared to non-progressors (*P *<0.001). We also compared the UFR of FST between subjects who were furosemide naïve versus those that were not; there was no difference between these (Additional file 1 - Table S5).

**Table 2:** Furosemide stress test effect on urine flow

Measurement time point	Combined	Non-progressors	Progressed to AKIN III	*p*
	*n *= 77	*n *= 52	*n *= 25	
**Hour 1**	251 (35.2)	329 (46.0)	89 (33.0)	0.001
**Hour 2**	296 (35.8)	392 (42.2)	96 (46.6)	0.001
**Hour 3**	246 (26.6)	311 (31.7)	109 (35.4)	0.001
**Hour 4**	207 (24.1)	265 (31.1)	88 (23.4)	0.001
**Hour 5**	175 (18.6)	219 (22.8)	83 (23.7)	0.001
**Hour 6**	155 (17.4)	194 (22.3)	75 (17.4)	0.001

Urine volume in ml is shown as mean (standard error). AKIN, Acute Kidney Injury Network; n, number of patients.

We tested various combinations of the UO intervals to assess which had the best discriminative capacity (Table 3). We found that the sum of the first 2 hours of UO after the FST had the highest AUC to predict the primary outcome (0.87 in both cohort 1 and cohort 2). We also assessed the sensitivity and specificity of various 2-hour urine volumes to predict the primary and secondary outcomes (Table 4). The 2-hour UO of 200 ml or less had the best sensitivity and specificity to predict the primary outcome.

**Table 3:** Furosemide stress test receiver operation characteristics for progression to AKIN Stage III

A			
**Urine output measurement time point**	**ROC AUCs**		
	**Cohort 1 **	**Cohort 2 **	**Combined**
	***n *= 23**	***n *= 54**	***n *= 77**

**One hour**	0.83 (0.11)	0.82 (0.07)	0.82 (0.05)
**Two hours**	0.87 (0.09)	0.87 (0.07)	0.87 (0.05)
**Three hours**	0.84 (0.09)	0.87 (0.07)	0.86 (0.05)
**Four hours**	0.85 (0.09)	0.87 (0.07)	0.86 (0.05)
**Five hours**	0.85 (0.09)	0.87 (0.07)	0.85 (0.05)
**Six hours**	0.85 (0.09)	0.86 (0.07)	0.85 (0.05)

**B Furosemide stress test receiver operation characteristics for progression to AKIN stage III or death**			

**Urine output measurement time point**	**ROC AUCs**		
	**Cohort 1 **	**Cohort 2 **	**Combined**
	***n *= 23**	***n *= 54**	***n *= 77**

**One hour**	0.86 (0.11)	0.74 (0.08)	0.79 (0.06)
**Two hours**	0.89 (0.09)	0.76 (0.08)	0.81 (0.06)
**Three hours**	0.87 (0.09)	0.76 (0.08)	0.80 (0.06)
**Four hours**	0.87 (0.09)	0.76 (0.08)	0.80 (0.06)
**Five hours**	0.88 (0.09)	0.77 (0.08)	0.81 (0.06)
**Six hours**	0.87 (0.09)	0.76 (0.08)	0.80 (0.06)

The outcome was Acute Kidney Injury Network (AKIN) stage III only within 14 days of the FST. One hour is the first hour after the furosemide stress test (FST), two hours is the sum of the first and second hour after the FST. All measurements shown were significant at *P *<0.01. ROC AUC, receiver operating characteristic area under the curve.

Primary outcome was Acute Kidney Injury Network (AKIN) stage III or death within 14 days of furosemide stress test (FST). One hour is the first hour after the FST, two hours is the sum of the first and second hour after the FST. All measurements shown were significant at *P *<0.01. ROC AUC, receiver operating characteristic area under the curve; n, number of patients.

**Table 4:** Sensitivity and specificity of two hour urine thresholds for progression to AKIN stage III

A		
	**Combined cohort**	
**Total urine output over 2 hours**	**Sensitivity**	**Specificity**

		
**≤100 ml**	90.2%	60.0%
**<200 ml**	87.1%	84.1%
**<300 ml**	85.3%	88.0%
**<400 ml**	66.7%	88.0%
**<500 ml**	50.5%	88.0%

**B Sensitivity and specificity of two hour urine thresholds for progression to AKIN III or death**		

	**Combined cohort**	
**Total urine output over two hours**	**Sensitivity**	**Specificity**

**<100 ml**	93.3%	53.2%
**<200 ml**	90%	74.2%
**<300 ml**	87.8%	77.4%
**<400 ml**	66.7%	77.4%
**<500 ml**	53.3%	77.4%

The primary outcome was Acute Kidney Injury Network (AKIN) stage III within 14 days of the furosemide stress test.

The primary outcome was Acute Kidney Injury Network (AKIN) stage III or death within 14 days of the furosemide stress test.

In this pilot study, we have demonstrated that the FST is feasible and well tolerated in critically ill patients with AKI. Furosemide administration can be associated with vasodilation and hypotension, but we did not observe any of these complications during our study. We took careful measures to decrease this potential adverse event by ensuring that the patients were deemed clinically well-resuscitated, and when appropriate received isovolemic replacement of UO with isotonic fluids. This may in part explain why we did not observe any adverse events.

The performance of the FST to predict the primary outcome was robust and consistent in both cohorts, with a range in ROC AUC of 0.82 to 0.87 (Table 3). Importantly, in comparison, the capacity of baseline UFR to predict the primary outcome had an ROC AUC of 0.71 to 0.76 (Additional file 1 - Table S2). This finding supports the concept that the FST offers important clinical information not captured by baseline UFR alone. In our study, the performance of the FST was comparable or exceeded the performance of several AKI biomarkers in predicting AKI progression [6,8,9]. We found the first 2-hour interval had the best predictive capacity (0.87), and this interval corresponds with the maximum UFR in response to the FST. When we assessed specific UO cutoffs we found that the 2-hour UO of 200 cc offered the best combination of sensitivity and specificity (87.1% and 84.1%, respectively). Because acute tubular necrosis causes intratubular obstruction and back leak, we were unsure whether the standard UO kinetics in response to furosemide would be similar to those seen in patients without renal disease [23,24]. Previous investigators have shown that in patients without AKI, the maximum diuretic effect of furosemide occurs within the first three hours [25]. We showed similar kinetics in our study (Figure 2). Patients who progressed to AKIN III compared to those that did not progress were similar in age, CV SOFA score, APACHE II score, and baseline eGFR, and had a similar incidence of comorbidity (Table 1). Nephrotoxic exposure and clinical phenotype was also similar in progressors and non-progressors (Table 1). Not surprisingly, the progressor group tended to have more patients with AKIN stage II, a lower baseline UFR, and a higher mean cast score prior to FST. In multivariable analyses, UO response to FST was still statistically associated with progression to AKIN stage III, even when these variables were placed into the model (Additional file 1 - Table S4).

The concept of using furosemide to evaluate AKI is not entirely new. In 1973, Baek and colleagues [26] assessed 15 patients who did not have clinically apparent AKI at that time, subjected them to a furosemide challenge, and then evaluated the patients' free water clearance (C_H2O_). They found that C_H2O _near zero and a poor response to furosemide signaled that 'acute renal failure was imminent'. In this modest sized study, the dose of furosemide was not standardized and the study did not report if the patients had early stage AKI or any evidence of AKI at all. Nonetheless, our findings confirm the findings of that original report. Moreover, clinicians regularly give patients with oliguric AKI a furosemide challenge. However, there has not been a standardized approach with fluid replacement, early assessment, and appropriate clinical cutoffs to guide care.

In this study, we have used the FST as a functional test to predict progressive AKI. Urine biomarkers have been used previously to predict worsening AKI. The predictive value of the FST compares favorably with other recent biomarker studies. Hall and colleagues determined the ability of urine neutrophil gelatinase associated lipocalin (NGAL), kidney injury molecule-1 (KIM-1) and IL-18 to predict worsening AKI (unadjusted AUC values were 0.71, 0.64 and 0.63 respectively) [27]. The TRIBE-AKI consortium found unadjusted AUC values of 0.63 for IL-18, and 0.58 and 0.74 for urinary and plasma NGAL, respectively [6]. Koyner and colleagues in a separate study found that π- glutathione S-transferase (GST) predicted progression to stage III AKI with an AUC of 0.86 [28]. We recently found that urinary angiotensinogen predicts worsening AKI with an AUC of 0.70 [17].

Although the findings in this study show good performance metrics for the FST, the use of the FST in patients who are not appropriately resuscitated can be potentially deleterious. We cannot overemphasize the point that patients need to be euvolemic before undertaking any type of furosemide challenge, and that volume replacement is mandatory in patients who are not obviously volume overloaded, as the mean UO in response to the challenge was over 1.3 L in 6 hours. In addition, the FST should be conducted in an appropriate clinical setting where UO, heart rate, and blood pressure can be monitored frequently.

The study has several limitations. Since this is a pilot study, larger more comprehensive studies of the FST are warranted in order to fully understand the advantages and disadvantages of this dynamic functional test. Although previous clinical trials of furosemide use in AKI have not shown any beneficial effect, we cannot be sure that the FST did not impact the natural history of AKI and therefore affect its predictive performance [29,30]. Some investigators have suggested that furosemide is protective in AKI because its administration may decrease tubular oxygen consumption, in which case its early administration in AKI would be protective [31]. Clinical trials using furosemide early in the course of AKI are underway and may help determine whether furosemide has a role in the treatment of AKI [32]. In addition, we did not specifically study patients with acute decompensated heart failure, nephrotic syndrome, or other patient populations with diuretic resistance. As such, we cannot be certain that the FST will perform similarly in those patient populations.

Similar to the approach used in acute coronary syndrome before the advances in thrombolytic therapy, the clinical syndrome of angina (that is, chest pain and dyspnea) was followed by a biomarker assessment (that is, creatine phosphosphokinase-MB) to further risk-assess the patient. For those patients who were confirmed with a biomarker, stress testing (for example, dobutamine or treadmill stress test) was used to confirm the presence of severe coronary artery disease. We believe that a similar process can begin in patients with AKI. Patients with renal angina [33] can be further assessed with AKI biomarkers. For those patients in whom AKI biomarkers confirm AKI, FST could be used to assess the severity and prognosis of AKI. Because indiscriminate use of loop diuretics can be harmful, appropriate resuscitation prior to FST is mandatory, and the FST should not be used as a primary screening diagnostic. We suggest that future studies to test this hypothesis be conducted.

## Conclusions

In summary, the FST is a novel dynamic functional assessment of tubular function that has good predictive capacity to identify those patients who will progress to advanced-stage AKI. Combinations of risk assessment, AKI biomarkers, and response to the FST may be used to help answer the important clinical question: 'When, or, should I start RRT in my patient with AKI?'

## Key Messages

• The furosemide stress test (FST) is feasible and well-tolerated in critically ill patients with early AKI.

• The performance of the FST to predict the primary outcome was robust and consistent in both cohorts, with a range in ROC AUC of 0.82 to 0.87.

• Patients should be euvolemic before undertaking any type of furosemide challenge, and volume replacement is mandatory in patients who are not obviously volume overloaded.

• FST should be conducted in an appropriate clinical setting where UO, heart rate, and blood pressure can be monitored frequently.

• FST is a novel dynamic functional assessment of tubular function that appears to have good predictive capacity to identify those patients who will progress to advanced-stage AKI. Further validation studies of the FST are warranted.

## Abbreviations

ABW: actual body weight; AKIN: Acute Kidney Injury Network; AKI: acute kidney injury; APACHE: Acute Physiology and Chronic Health Evaluation; AUC: area under the curve; CHF: congestive heart failure; CKD: chronic kidney disease; CV: cardiovascular; DM: diabetes mellitus;eGFR: estimated glomerular filtration rate; FeNa: fractional excretion of sodium; FST: furosemide stress test; GW USS: George Washington University Urine Sediment Score; hOAT: human organic acid transporter; HTN: hypertension; IL-18: interleukin 18; IBW: ideal body weight; KIM-1: kidney injury molecule -1; NGAL: neutrophil gelatinase associated lipocalin; OR: odds ratio; π-GST: π-glutathione S-transferase; RRT: renal replacement therapy; ROC: receiver operating characteristic; SAKInet: Southern Acute Kidney Injury Network; Scr: serum creatinine; SOFA: Sequential Organ Failure Assessment; UFR: urinary flow rate; UO: urine output.

## Competing interests

LSC has links with Abbott Medical, Astute Medical, Alere Medical, Gambro Medical and Nxstage Medical. DLD has links with Astute Medical. JLK has links with Astute Medical, Abbott Medical, and Argutus Medical. ADS has links with Astute Medical. JAT has links with Astute Medical. All other authors declare that they have no competing interests.

## Authors' contributions

All authors read and approved the final manuscript. LSC conceived the study, helped conduct the trial and the literature search, made the Figures, collected data, performed data analysis and interpretation, and wrote the manuscript. DLD helped conduct the trial, collect data, perform the literature search, data analysis and interpretation, and wrote the manuscript. EBM helped conduct the trial, collect data, perform data analysis and interpretation, and wrote the manuscript. JLK helped conduct the trial, collected data, data analysis, interpretation, and wrote the manuscript. JMA did data analysis, literature search, interpretation, and wrote the manuscript. ADS did data analysis, interpretation, and wrote the manuscript. JAT did data analysis, interpretation, and wrote the manuscript. SAT helped conduct the trial, collected data, and performed data analysis. PLK did the literature search, data analysis and interpretation, and wrote the manuscript. MGS did data analysis and interpretation, and wrote the manuscript.

## Supplementary Material

Additional file 1 **Supplementary Methods, Table S1 to S5**. Click here for file
