# Supplementary material for: Development and Standardization of a Furosemide Stress Test to Predict the Severity of Acute Kidney Injury
Source: Crit Care. 2013 Sep 20;17(5):R207. doi: 10.1186/cc13015 (PMC4057505; doi:10.1186/cc13015)
Supplement: Additional file 1 — Supplementary Methods, Table S1 to S5. [file cc13015-S1.DOC]

**Additional file 1 – Methods, Table S1 to Table S5**

**Methods**

Fractional excretion of sodium was calculated as described in Bagshaw and colleagues.[^1^](#_ENREF_1) MDRD equation was used to calculate the eGFR as described in Levey and colleagues.[^2^](#_ENREF_2) Cardiovascular SOFA score and APACHE II score were calculated as previously described.

**Table S1, Acute Kidney Injury Network Criteria**

| **Stage** | **Serum Creatinine Criteria** | **Urine Output Criteria** |
| --- | --- | --- |
|  |  |  |
| **I** | Increase in serum creatinine of >0.3 mg/dL or increase to > 150- 200% from baseline | < 0.5 ml/kg/hr for more than 6 hours |
| **II** | Increase in serum creatinine to > 200- 300% from baseline | < 0.5 ml/kg/hr for more than 12 hours |
| **III** | Increase in serum creatinine to > 300% from baseline (or serum creatinine of > 4.0 mg/dL with an acute increase of at least 0.5 mg/dL) or need for renal replacement therapy | 0.3 ml/kg/hr for 24 hours or anuria for 12 hours |

Table S2, Furosemide Stress Test and Urinary Flow Rate Receiver Operation

Characteristics for Progression to AKIN Stage III

Variable ROC AUC S.E.

| UFR-raw | 0.76 | 0.08 |
| --- | --- | --- |
| UFR—IBW | 0.71 | 0.09 |
| UFR-ABW | 0.76 | 0.08 |
| Two Hour UO | 0.87 | 0.08 |

Legend: UFR-raw = absolute values urinary flow rate (UFR), UFR-IBW = the UFR divided by ideal body weight, UFR-ABW = UFR divided by actual body weight, Two hour UO = total urine output for the two hour after FST.

**Table S3, Patient Characteristics Between Cohorts**

| **Variable** |  | **Combined** |  | **Cohort 1** | **Cohort 2** |  | **p** |
| --- | --- | --- | --- | --- | --- | --- | --- |
|  |  | *n = 77* |  | *n = 23* | *n = 54* |  |  |
| **Age (years, s.e.)** |  | 65.3 (1.6) |  | 70.0(2.5) | 63.1(2.0) |  | 0.03 |
| **Male (n,%)** |  | 33(42.8%) |  | 11(47.8%) | 22(40.7%) |  | 0.62 |
| **Race** |  |  |  |  |  |  |  |
| **African American** |  | 44 (57.1%) |  | 12 (52.2%) | 32(59.3%) |  | 0.57 |
| **Caucasian** |  | 23(29.9%) |  | 7(30.4%) | 16(29.6%) |  | 0.90 |
| **Hispanic** |  | 10(13.0%) |  | 4(17.4%) | 6(11.1%) |  | 1.00 |
|  |  |  |  |  |  |  |  |
| **Comorbidites n (%)** |  |  |  |  |  |  |  |
| **CKD** |  | 24 (31%) |  | 6(26.1%) | 18(33.3%) |  | 0.60 |
| **Hypertension** |  | 60 (78%) |  | 19(82.6%) | 41(75.9%) |  | 0.77 |
| **CHF** |  | 25(33%) |  | 8(34.8%) | 17(31.5%) |  | 0.49 |
| **DM** |  | 35(44%) |  | 11(4.8%) | 24(44.4%) |  | 0.49 |
|  |  |  |  |  |  |  |  |
| **Nephrotoxic Exposures** |  |  |  |  |  |  |  |
| **NSAIDS** |  | 8(10)% |  | 0(0%) | 8(14.8%) |  | 0.10 |
| **Aminoglycosides** |  | 1(1%) |  | 0(0%) | 1(1.9%) |  | 1.00 |
| **Amphotericin** |  | 2(3%) |  | 0(0%) | 2(3.7%) |  | 1.00 |
| **Contrast** |  | 21(27%) |  | 7(30.4%) | 14(25.9%) |  | 0.78 |
| **Post-cardiac surgery** |  | 9(11.7%) |  | 0(0%) | 9(16.7%) |  | 0.05 |
| **Sepsis** |  | 15(19.5%) |  | 0(0%) | 15(27.8%) |  | 0.01 |
|  |  |  |  |  |  |  |  |
|  |  |  |  |  |  |  |  |
| **Clinical Data** |  |  |  |  |  |  |  |
| **Baseline eGFR (ml/min/1.73m^2^)** |  | 68.6 (4.1) |  | 58(9.7) | 73.2(4.0) |  | 0.16 |
| **Furosemide Naive** |  | 29 (37.7%) |  | 8(34.8%) | 21(38.9%) |  | 0.80 |
| **Urine Cast Score** |  | 2.3 (.13) |  | 2.2(0.27) | 2.4 (0.15) |  | 0.57 |
| **FeNa (above 1%)*** |  | 14 (18%) |  | 4(17.4%) | 10(18.5%) |  | 0.91 |
| **CV SOFA Score** |  | 1.16 (0.3) |  | 1.9(0.29) | 1.18(0.19) |  | 0.04 |
| **APACHE II Score** |  | 17.8 (1.11) |  | 21.3(1.7) | 17.8(1.1) |  | 0.09 |
|  |  |  |  |  |  |  |  |
| AKIN Stage at Enrollment |  |  |  |  |  |  |  |
| **AKIN I** |  | 41(53.%) |  | 14(60.9%) | 27(50%) |  | 1.00 |
| **AKIN II** |  | 36(46.7%) |  | 9(39.18%) | 27(50%) |  |  |
|  |  |  |  |  |  |  |  |
| **Outcomes** |  |  |  |  |  |  |  |
| **Death** |  | 16(20.7%) |  | 8(34.8%) | 8(14.8%) |  | 0.07 |
| **AKIN Stage III** |  | 25 (32.4%) |  | 11(47.8%) | 14(25.9%) |  | 0.07 |
| **RRT** |  | 11 (14.2%) |  | 5(21.7%) | 6(11.1%) |  | 0.29 |
| **Death/AKIN III** |  | 32 (41.6 %) |  | 13(56.5%) | 19(35.2%) |  | 0.13 |

Legend: All data presented as mean +s.e. unless otherwise indicated. CKD = chronic kidney disease, CHF = congestive heart failure, DM= diabetes mellitus, NSAIDs = non-steroidal anti-inflammatory drugs, CV SOFA = cardiovascular sequential organ failure assessment, APACHE = acute physiology and chronic health evaluation, FeNa = fractional excretion of sodium, RRT = renal replacement therapy. RPP = renal perfusion pressure.

*FeNa not assessed on 29 patients since GW USS already >2 at the time of assessment

Table S4a, Multivariable Logistic Regression for Progression to AKIN Stage III

| **Variable** |  | **Odds Ratio** |  | **95% CI** | **p value** |
| --- | --- | --- | --- | --- | --- |
| **APACHE II Score** |  | 1.05 |  | 0.97 – 1.13 | 0.25 |
| ***Two Hour UO*** |  | *0.97* |  | *0.95-0.99* | *0.02* |
| **Baseline eGFR** |  | 0.99 |  | 0.97-1.01 | 0.45 |
| **AKIN Stage II** |  | 4.1 |  | 1.1 -14.1 | 0.03 |

Legend: Two hour UO is for each 10cc of UO in increase. APACHE II Score includes age as a part of the score. Baseline eGFR = MDRD calculated eGFR.

Table S4b, Multivariable Logistic Regression for Progression to AKIN Stage III

| **Variable** |  | **Odds Ratio** |  | **95% CI** | **p value** |
| --- | --- | --- | --- | --- | --- |
|  |  |  |  |  |  |
| ***Two Hour UO*** |  | *0.98* |  | *0.96 -0.99* | *0.05* |
| **GW USS Cast Score** |  | 1.08 |  | 0.62 -1.9 | 0.79 |
| **APACHE II Score** |  | 1.1 |  | 0.96 -1.1 | 0.42 |
| **AKIN Stage II** |  | 3.0 |  | 0.86 -10.5 | 0.09 |
| **Baseline UFR** |  | 0.98 |  | 0.95-1.01 | 0.07 |

Legend: Two hour UO is for each 10cc of UO in increase. GW USS = George Washington Urinary Sediment Score. UFR = urine flow

Legend: Two hour UO is for each 10cc of UO in increase. GW USS = George Washington Urinary Sediment Score.

Table S4c, Backward Elimination Logistic Regression to Predict Progression to AKIN III

| **Variable** |  | **Odds Ratio** |  | **95% CI** | **p value** |
| --- | --- | --- | --- | --- | --- |
|  |  |  |  |  |  |
| ***Two Hour UO*** |  | *0.98* |  | *0.96-0.99* | *0.04* |
| **AKIN Stage II** |  | 4.7 |  | 1.7-12.8 | 0.003 |
| **Baseline UFR** |  | 0.98 |  | 0.97-1.01 | 0.08 |

Legend: Age, APACHE II score, CV SOFA score, baseline eGFR, post-cardiac surgery, sepsis, GW USS cast score, CHF, DM, HTN, AKIN stage II, Baseline UFR, and Two Hour UO placed into a backward elimination logistic regression. Remaining covariates shown in Table above.

Two hour UO is for each 10cc of UO in increase. GW USS = George Washington Urinary Sediment Score, UFR = urine flow rate (ml/hr), CHF = history of congestive heart failure, DM = history of diabetes mellitus, and HTN = history of hypertension.

Legend: Two hour UO is for each 10cc of UO in increase. GW USS = George Washington Urinary Sediment Score.

Table S5, Furosemide Stress Test Effect on Urine Flow

| **Time Period** |  | **Combined** |  | **Furosemide Naive** | **Non-naive** |  | **p** |
| --- | --- | --- | --- | --- | --- | --- | --- |
|  |  |  |  |  |  |  |  |
|  |  | *n =77* |  | *n= 29* | *N = 48* |  |  |
|  |  |  |  |  |  |  |  |
| **Hour 1 (ml)** |  | 251 (35.2) |  | 331 (68.5) | 258 (37.2) |  | 0.11 |
| **Hour 2 (ml)** |  | 296 (35.8) |  | 348 (66.6) | 264 (40.9) |  | 0.29 |
| **Hour 3 (ml)** |  | 246 (26.6) |  | 285 (52.1) | 223 (28.6) |  | 0.30 |
| **Hour 4 (ml)** |  | 207 (24.1) |  | 240 (48.6) | 188 (25.4) |  | 0.35 |
| **Hour 5 (ml)** |  | 175 (18.6) |  | 204 (35.9) | 157 (22.5) |  | 0.26 |
| **Hour 6 (ml)** |  | 155 (17.4) |  | 167 (33.6) | 147 (19.6) |  | 0.61 |

Legend: Urine volumes shown as mean (s.e.).

**Additional file references**

1. Bagshaw SM, Bennett M, Devarajan P, Bellomo R. Urine biochemistry in septic and non-septic acute kidney injury: a prospective observational study. J Crit Care 2012.

2. Levey AS, Bosch JP, Lewis JB, Greene T, Rogers N, Roth D. A more accurate method to estimate glomerular filtration rate from serum creatinine: a new prediction equation. Modification of Diet in Renal Disease Study Group. Ann Intern Med 1999;130:461-70.

3. Vincent JL, Moreno R, Takala J, et al. The SOFA (Sepsis-related Organ Failure Assessment) score to describe organ dysfunction/failure. On behalf of the Working Group on Sepsis-Related Problems of the European Society of Intensive Care Medicine. Intensive Care Med 1996;22:707-10.

4. Knaus WA, Draper EA, Wagner DP, Zimmerman JE. APACHE II: a severity of disease classification system. Crit Care Med 1985;13:818-29.
